# Functional Properties of Low-Modulus PMMA Bone Cements Containing Linoleic Acid

**DOI:** 10.3390/jfb12010005

**Published:** 2021-01-17

**Authors:** Céline Robo, David Wenner, S. J. Kumari A. Ubhayasekera, Jöns Hilborn, Caroline Öhman-Mägi, Cecilia Persson

**Affiliations:** 1 Department of Materials Science and Engineering, Division of Applied Materials Science, Uppsala University, 751 21 Uppsala, Sweden; celine.robo@orange.fr (C.R.); david.wenner@hotmail.com (D.W.); caroline.ohman@angstrom.uu.se (C.Ö.-M.); 2 Department of Chemistry-BMC, Analytical Chemistry, Uppsala University, 751 23 Uppsala, Sweden; Kumari.Ubhayasekera@kemi.uu.se; 3 Department of Chemistry, Division of Polymer Chemistry, Uppsala University, 751 21 Uppsala, Sweden; Jons.Hilborn@kemi.uu.se

**Keywords:** PMMA bone cement, low-modulus, mechanical properties, bending, sterilization, screw pull-out, vertebroplasty, kyphoplasty

## Abstract

Acrylic bone cements modified with linoleic acid are a promising low-modulus alternative to traditional high-modulus bone cements. However, several key properties remain unexplored, including the effect of autoclave sterilization and the potential use of low-modulus cements in other applications than vertebral augmentation. In this work, we evaluate the effect of sterilization on the structure and stability of linoleic acid, as well as in the handling properties, glass transition temperature, mechanical properties, and screw augmentation potential of low-modulus cement containing the fatty acid. Neither ^1^H NMR nor SFC-MS/MS analysis showed any detectable differences in autoclaved linoleic acid compared to fresh one. The peak polymerization temperature of the low-modulus cement was much lower (28–30 °C) than that of the high-modulus cement (67 °C), whereas the setting time remained comparable (20–25 min). The T_g_ of the low-modulus cement was lower (75–78 °C) than that of the high-stiffness cement (103 °C). It was shown that sterilization of linoleic acid by autoclaving did not significantly affect the functional properties of low-modulus PMMA bone cement, making the component suitable for sterile production. Ultimately, the low-modulus cement exhibited handling and mechanical properties that more closely match those of osteoporotic vertebral bone with a screw holding capacity of under 2000 N, making it a promising alternative for use in combination with orthopedic hardware in applications where high-stiffness augmentation materials can result in undesired effects.

## 1. Introduction

Acrylic bone cement, based on poly(methyl methacrylate) (PMMA) has become a popular biomaterial in the field of orthopaedics, since its first use in fixating a hip joint prostheses in the 1950s [1,2]. PMMA bone cement has also been widely used in other orthopaedic applications, particularly for the treatment of vertebral compression fractures (VCFs) [3]. VCFs are likely to occur in patients affected by osteoporosis, a condition characterized by microarchitectural deterioration of bone tissue and low bone mass [4]. Vertebroplasty (VP) is a common surgical intervention used to relieve back pain attributed to VCFs, and involves injecting a bone cement within the fractured vertebrae under real-time fluoroscopic image guidance. This minimally invasive procedure has proven to be a generally successful solution [5,6,7], despite possible complications related to the use of the cement. The bone cement sets via an exothermic polymerization reaction during which heat is generated (with a temperature up to 100–122 °C) [8,9], in some cases causing thermally induced necrosis of the surrounding bone tissue [8,10,11]. In addition, unreacted monomer residing after polymerization could be toxic and induce necrosis of the surrounding soft tissue [12,13]. The main concerns are however the risk of cement leakage during the VP procedure [14,15,16]—which has been reported to cause adverse effects ranging from nerve root compression to pulmonary embolisms in rare cases—and the possible contribution to the development of additional adjacent VCFs [16,17,18,19]. In fact, it has been suggested that vertebral augmentation with stiff PMMA bone cement may facilitate additional osteoporotic fractures in the vicinity of the treated vertebrae [20,21,22,23]. Although additional fractures are likely to appear due to the natural course of osteoporosis, the disproportionally high number of new fractures occurring next to the treated vertebrae [24,25] suggest that the high cement stiffness [26,27,28,29], and/or high volume fill [29,30,31] may facilitate new fractures. Indeed, ex vivo and finite element studies have reported an increase of the overall vertebral body stiffness of 13% to 33% due to the injection of PMMA bone cement [26,27,32,33]. The increase in pressure on the endplates adjacent to the augmented vertebrae may alter the natural stress state and result in higher risks of new fractures in the vicinity.

New formulations of less stiff bone cement, i.e., with a reduced Young’s modulus, have therefore been synthesized by either creating micropores in the polymer matrix [34,35,36,37] or by incorporating various additives, such as organic plasticizers [38,39,40] or elastomeric nanoparticles [41] into the PMMA formulation. Others have suggested using bone cement based on poly(ethylmethacrylate-*co*-n-butylmethacrylate) monomers (PEMA-nBMA) [42,43]. More recently, the use of fatty acids (FA) and triglyceride oils has shown promising results in reducing the stiffness of PMMA bone cement [26,39,44,45,46,47]. In particular, López et al. [45] added 12 wt% Castor oil into the vertebroplastic bone cement Osteopal^®^V (Heraeus Medical GmbH, Hanau, Germany) and reported a reduction in strength and elastic modulus of 83% and 70%, respectively. However, in vitro studies revealed that using large amounts of this oil could lead to an adverse effect on the cytocompatibility of the cements [45,47]. On the other hand, fatty acid-modified bone cements containing linoleic acid (LA) have shown to exhibit adequate in vitro and in vivo responses [44,46], as well as bone-compliant mechanical properties [44,46]. However, earlier work was limited to mechanical tests in compression, and to the best of the authors’ knowledge, there are no reports available on the flexural properties and screw holding power of the low-modulus LA-modified cement. When the cement is used for screw augmentation and/or when already present in the vertebrae, and there is a need for hardware insertion, these properties would of course be highly relevant. Therefore, one aim of the current study was to determine the bending modulus, bending strength, and pull-out force of the LA-modified cement.

Furthermore, cement components need to be sterile prior to implantation. While the effect of the sterilization process on standard acrylic bone cement has been described in the literature [48,49], there is no data available on the functional properties of sterilized low-modulus LA-modified PMMA bone cement. A second aim of this study was hence to investigate the effect of sterilization on LA composition, as well as to evaluate the subsequent functional properties of the sterilized formulation in terms of quasi-static mechanical properties and handling properties.

## 2. Materials and Methods 

### 2.1. Material Preparation

A commercially available PMMA bone cement intended for VP, V-Steady^TM^ (G21 Srl, San Possidonio, Italy), was used and modified with 9-*cis*,12-*cis*-linoleic acid (LA, ≥99%, Sigma-Aldrich, St. Louis, MO, USA). The cement powder contains poly(methyl methacrylate), benzoyl peroxide and zirconium dioxide, and the liquid consists mainly of methyl methacrylate and small amounts of N,N-dimethyl-*p*-toluidine and hydroquinone. Unlike the base cement, LA was delivered in a non-sterile vial and was therefore autoclaved at 121 °C for 20 min (Systec VX-95, Systec GmbH, Linden, Germany). The unmodified cement was prepared as recommended by the manufacturer by mixing the powder and liquid components manually in a glass mortar with a spatula for 30 to 45 s at room temperature. The modified bone cements (containing sterile or non-sterile LA) were prepared as described elsewhere [44], by first pre-mixing 12 vol% LA with the liquid phase for a few seconds in a vortex-genie 2 mixer (Scientific Industries, Bohemia, NY, USA). The monomer phase was then added to the powder phase and mixed manually. The resulting paste was poured into moulds for direct analysis or allowed to set in phosphate buffered saline solution (PBS tablets, P4417, Sigma-Aldrich, Merck, Darmstadt, Germany) at 37 °C for differing periods of time (see below). In this study, the sample designations VS, VSLA-S and VSLA-NS refer to unmodified cement (control cement, V-Steady^TM^), sterile LA-modified cement, and non-sterile LA-modified cement, respectively.

### 2.2. ^1^H NMR Spectroscopy

The chemical structure of sterilised and non-sterilised LA were analysed by proton nuclear magnetic resonance spectrometry (^1^H NMR) and spectra were obtained under ambient conditions on a Jeol ECP-400 NMR (JEOL, Tokyo, Japan) using deuterated chloroform (CDCl_3_) as a solvent. Chemical shifts were referenced towards the residual solvent peak and were in ppm. Coupling constants were in Hz. This test was run in triplicates of each group. 

### 2.3. Supercritical Fluid Chromatography-Tandem Mass Spectrometry (SFC-MS/MS)

A validated protocol for analysis of free fatty acids was executed for the determination of linoleic acid (LA, C18:2) in autoclaved sample. The effect of autoclaving on LA was analysed with Ultra Performance Supercritical Fluid Chromatography (UPSFC-MS/MS, Waters ACQUITY^®^ UPC^2^™, Milford, MA, USA) coupled with tandem mass spectrometry (XEVO^®^ TQ-S, Milford, MA, USA). Chromatographic separation was achieved on a HSS C18 SB column (100 mm 3.0 mm, 1.7 µm, Waters, Milford, MA, USA) at 40 °C. The mobile phase flow rate was maintained at 1.0 mL/min with a gradient elution (eluent A, CO_2_ (99.99%); eluent B, methanol with 10 mM of ammonium acetate). The gradient program was started with 3% of component B, then a linear gradient of B was programmed from 6% to 10% for 0.2–1 min, followed by a linear gradient up to 25% B for 2–3 min. Finally, the linear gradient step was set down to 5% in 3 min. Mass spectrometric detection was performed using electrospray ionisation in the negative ionization mode (ESI^−^) with unit mass resolution with nitrogen and argon serving as desolvation and collision gas, respectively. The data acquisition range was *m/z* 50–500. The collision energy and cone voltage were optimized for the compound to generate the most abundant deprotonated product ions of LA ([M-H]^−^ with *m/z* = 279.11 Da). Identification was based on the multiple reactions monitoring (MRM) transition of linoleic acid. All data were acquired and processed using Mass Lynx^TM^ 4.1 software (Waters, Milford, MA, USA). Triplicate analyses of each sample were carried out and the average values were reported (CV < 3%).

### 2.4. Handling Properties

The setting time (*t_setting_*), doughing time (*t_doughing_*), and maximum polymerization temperature (*T_max_*) were determined from the temperature-versus-time plot, as described in ISO 5833 [50]. Freshly mixed cement doughs were transferred into 3 mL syringes (barrel diameter of 8.55 mm, outlet diameter of 1.90 mm) to be injected in air through a 13 G needle (13 G, inner Ø = 1.8 mm, outer Ø 2.4 mm, 10 cm long). The injectability test was started 5 min after start of mixing at room temperature (21 °C), since according to the manufacturer, this is when the “application phase” of the cement begins. The cements were extruded at slow (1.5 mm/min) and moderate (5.0 mm/min) injection rates because an optimal filling of a vertebra might depend on the injection pressure and rate used by the clinician [51,52,53,54], as well as other factors. Extrusion of the paste was stopped when a force of 150 N was reached, this limit being a value approaching the physical force that a clinician can apply during vertebroplasty [34,54]. This test was performed in triplicate for each injection rate.

### 2.5. Differential Scanning Calorimetry 

The glass transition temperature (T*_g_*) of the cements was determined using differential scanning calorimetry (DSC Q1000, TA instruments, New Castle, DE, USA). Freshly mixed cements were transferred into aluminium pans hermetically sealed with a lid. The first measurements were performed within the first 24 h after mixing started, and the second measurements were carried out after the specimens had set for 2 weeks in the aluminium pan/lid at 37 °C. A heat/cool/heat ramp experiment was carried out from −10 °C to 200 °C at a rate of 10 °C/min. The T*_g_* was determined from the second heat ramp. These tests were performed in triplicates for each group.

### 2.6. Mechanical Testing 

The compressive, flexural, and pull-out properties of the cements were determined using a universal testing machine (Shimadzu, AGS-X, Kyoto, Japan). Cylindrical specimens of 6 mm in diameter and 12 mm in height were used for quasi-static mechanical testing in compression. Specimen compression was performed at a crosshead speed of 20 mm/min according to ISO 5833 [50]. Quasi-static mechanical properties of the cements were determined after storage in PBS (37 °C) for 24 h, 2 and 4 weeks. The compressive elastic modulus (E_c_) and compressive strength (σ_CS_) of six specimens (per time point) were determined from the load versus-displacement curves. In accordance with the standard, the compressive strength was calculated from the 2% offset load or the upper yield-point load, whichever occurred first. Stiffness measurements were corrected for machine compliance.

Based on the results from Section 3.1, Section 3.2, Section 3.3 and Section 3.4 (see below) and the compressive tests, the remaining mechanical testing was performed only on vs. and VSLA-NS. Rectangular specimens (length 75 mm, width 10 mm, depth 3.3 mm) were used to determine the bending strength and bending modulus. Bending tests were carried out using a 4-point set up and cements were deformed at a crosshead speed of 5 mm/min as according to ISO 5833 [50]. Prior to testing, the cements were stored for 24 h, 2, 4, and 6 weeks in PBS (37 °C). Bending modulus (E*_b_*) and bending strength (σ*_b_*) were calculated according to ISO 5833, using the force at break of the samples. The E*_b_* for unbroken samples was calculated from the slope of the force-displacement curve, using the maximum load reached by the samples before bending. Five specimens per time point were evaluated for this test.

Cylindrical blocks of PMMA bone cement (20 mm diameter and 20 mm height) were prepared and allowed to set for 2 weeks in PBS (37 °C) before insertion of half threaded cancellous screws (titanium orthopaedic cancellous screws, HB 6.5, Jiangsu IDEAL Medical Science & Technology, Zhangjiagang, China). A pilot hole of 3.2 mm is usually recommended for cancellous screws of 6.5 mm outer diameter [55]. However, preliminary tests revealed that the insertion of screws into pilot holes as small as the one recommended was not possible, as the material was too hard. Therefore, the diameter of the pilot hole was increased to 3.8 mm and holes were drilled in the centre of the test block to a depth of 15 mm. A pull-out force was applied along the long axis of the screws at a crosshead speed of 5 mm/min according to ASTM F543-07 [56] and the maximum pull-out force was recorded. 

The holding power of the screws was also evaluated in polyurethane Sawbone^®^ cubes (blocks of 30 × 30 × 40 mm, 0.24 g/cm^3^ purchased from Sawbones^®^ Europe AB, Malmö, Sweden) to mimic the injection of the cement into healthy cancellous bone. Pilot holes of diameter 3.2 mm and depth 20 mm were created in the centre of each block. Approximately 1 mL of cement paste was injected into the pilot hole before insertion of the cancellous screws. Bovine cortical shells (discs of 2.5–3.5 mm thickness, with a 8 mm hole in the centre) were attached to the surface of the Sawbone^®^ (containing the screw in the centre) with additional cement, to mimic the in vivo scenario with the presence of trabecular bone and a cortical shell, as illustrated in Pujari-Palmer et al. in their Figure 1D [57]. The system was allowed to set for 24 h at room temperature before testing. Pull-out tests were carried out as previously described using a displacement rate of 1 mm/min and the maximum pull-out force was recorded.

### 2.7. Statistical Analysis 

Statistical analysis was performed in IBM SPSS Statistics V. 22 (IBM, Chicago, IL, USA) using a one-way ANOVA followed by a Scheffe’s post-hoc to evaluate statistical differences between the materials. A significance level of α = 0.05 was used. 

## 3. Results

### 3.1. ^1^H NMR Analysis

**Figure 1:**
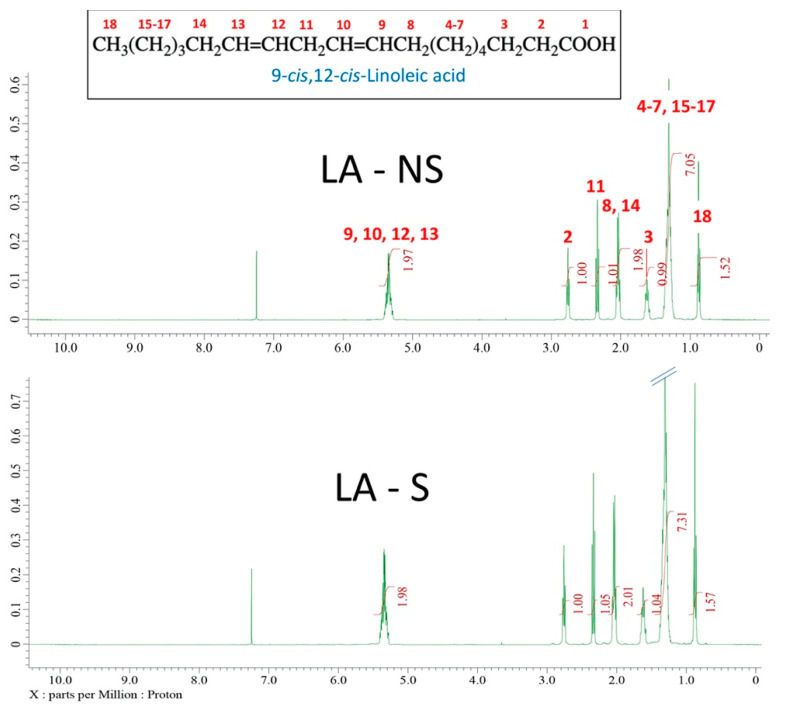
^1^H NMR spectra of non-sterilized LA (LA-NS) and sterilized (LA-S). Only one spectrum per group is plotted in the figure.

The ^1^H NMR spectra and chemical shift values of non-sterilized LA (LA-NS) and sterilized (LA-S) with peak assignments are presented in Figure 1. The analysis revealed no detectable differences between LA-S and LA-NS. The spectra confirmed the chemical composition of pure 9-*cis*-12-*cis*-linoleic acid and is matched by identification in the literature [58]. ^1^H NMR (C_6_D_6_, 400 MHz): δ 0.88 (t, 2H), 1.33 (m, 14H), 1.62 (quint, 2H), 2.01 (q, 4H), 2.36 (t, 2H), 2.76 (t, 2H), 5.33 (m, 4H), residual internal C6D5H (δ 7.26). 

### 3.2. SFC-MS/MS Analysis

**Figure 2:**
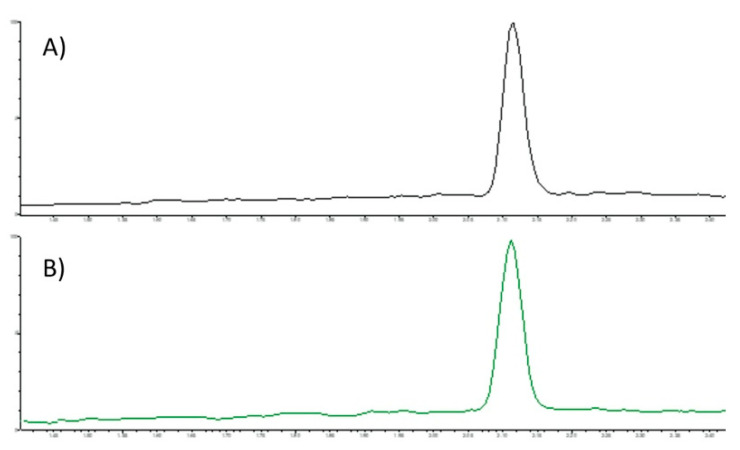
Example chromatogram of alpha linoleic acid (C18:2) from Standard (**A**) and Autoclaved standard (**B**).

The areas under each peak characteristic of LA (C18:2) were measured and a mean was calculated for each group (LA-S and LA-NS, n = 3). No major change between the groups could be detected. No other signals were detected in the SFC elution pattern of LA: the SFC full scan chromatogram was clean and baseline separation was achieved as a signal in the MRM chromatogram of LA, as shown in Figure 2. 

### 3.3. Handling Properties

The handling properties of the vs. cement and the modified cements are shown in Table 1. The use of LA (sterilized and non-sterilized) in the cement did not significantly modify the setting time of the vs. cement (*p* > 0.06) but sterilized cements had statistically lower setting time than non-sterilized cements (*p* = 0.01). 

The maximum polymerization temperature of the vs. cement was reduced approx. by half (*p* < 0.001) when LA was added. There was no difference between the peak temperatures measured for VSLA-NS and VSLA-S cements (*p* = 0.19). The addition of LA slightly but significantly increased the doughing time of regular cements (*p* < 0.03).

**Figure 3:**
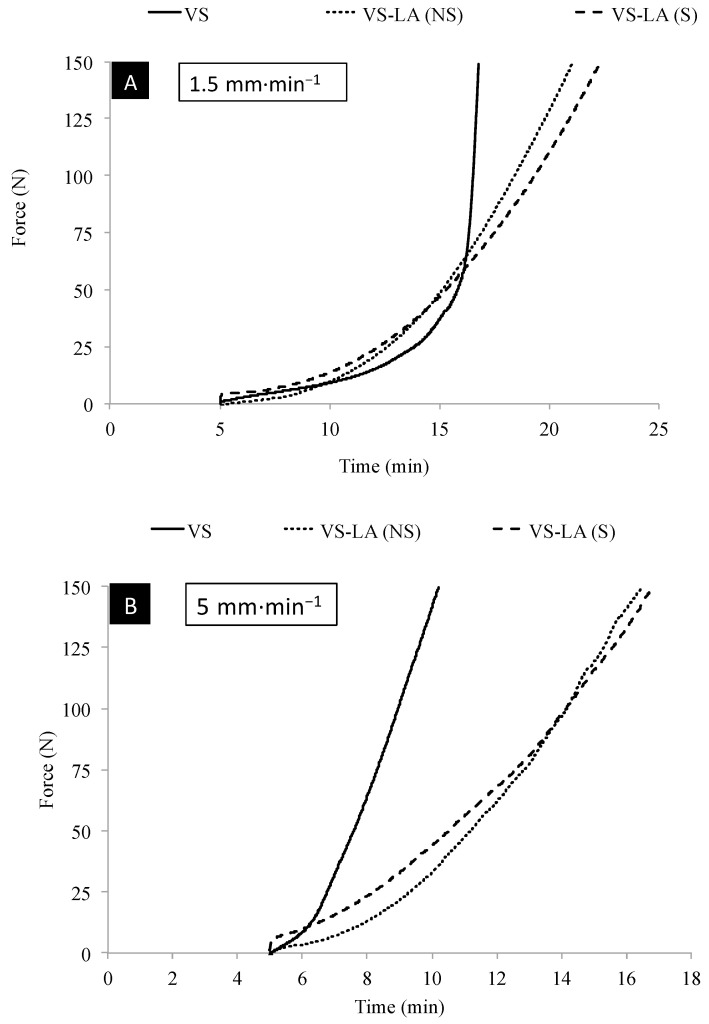
Injectability curves of the control (VS) and LA-modified cements (sterilized and non-sterilized LA) when injected at a crosshead speed of (**A**) 1.5 mm·min^−1^ and (**B**) 5 mm·min^−1^; n = 3 for each group but only one (typical) specimen per group and speed of injection is shown in the figure.

The injectability of the cement was affected by the addition of LA (sterilized and non-sterilized), and the modified cements could be injected during longer times, as seen in Figure 2. All pastes were injected without any observable phase separation. VSLA-S and VSLA-NS cements could be extruded at low (1.5 mm·min^−1^) and high speed (5 mm·min^−1^) until reaching the limit of 150 N, and were still manually injectable after the end of the test. The injection speed and sterilization process had no significant effect on the injectability (in terms of % injected material) of these cements (Figure 3) and the LA-modified cements could be injected approx. during 16 min and 20 min at high and low speed, respectively. vs. cement could be injected at low (except for one case) and high speed until reaching the limit of 150 N. The cement was still manually injectable after the end of the test only at high speed and the injectability (in terms of % injected material) gradually decreased as the speed of injection was reduced.

### 3.4. DSC Analysis

The glass transition temperatures at two weeks were significantly higher (*p* < 0.04) than those measured at 24 h for all cements, as seen in Table 1. The T*_g_* of LA-modified (sterilized and non-sterilized) cements were significantly lower than the T*_g_* of vs. cement (*p* < 0.001). There was no statistical difference between the T*_g_* of VSLA-NS and VSLA-S (*p* > 0.83).

### 3.5. Mechanical Testing 

**Figure 4:**
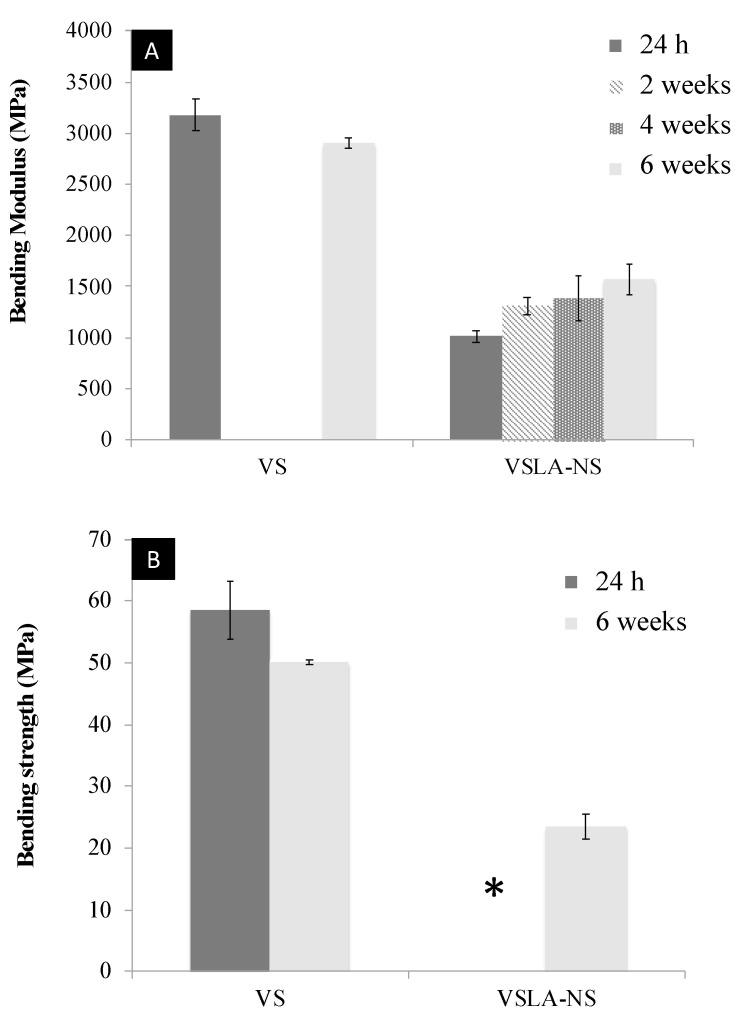
Flexural mechanical properties: (**A**) Bending modulus and (**B**) Bending strength of the control (VS) and non-sterilized LA-modified cements (VSLA-NS); n = 5 for each group and time point. The “*” indicates the samples that did not break.

**Figure 5:**
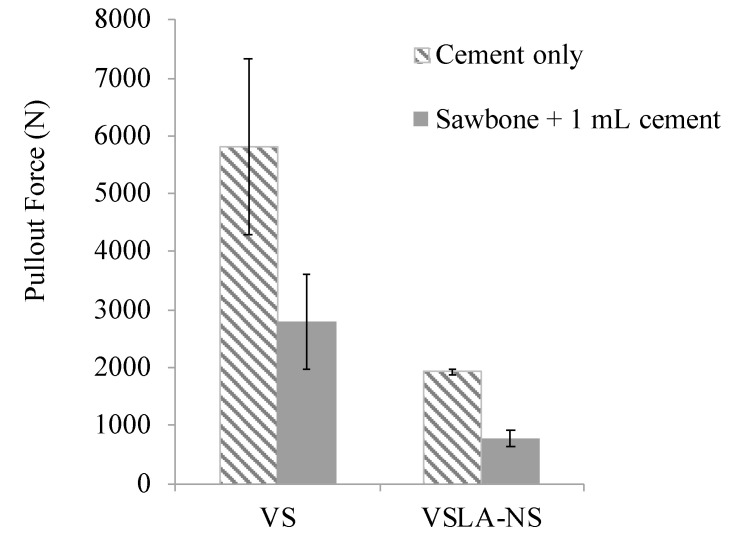
Pullout force of vs. and VSLA-NS augmented screws from cement only (pilot hole of 3.8 mm; n = 5) and from Sawbone^®^ (pilot hole of 3.2 mm; n = 8). The pull-out force from Sawbone^®^ only is 36.3 ± 8.4 N [57].

Data from the quasi-static mechanical tests are presented in Table 2, Figure 4 and Figure 5. The mechanical properties of the vs. cement remained similar over time (Table 2), with no statistically significant differences in elastic modulus (*p* > 0.98) or strength (*p* > 0.65) among the different time points. The mechanical properties of both types of LA-modified cement (VSLA-S and VSLA-NS) were significantly different compared to the control after 24 h of setting, with a decrease of stiffness and strength up to 70–80% (*p* < 0.001). The stiffness of VSLA-S increased progressively from 559.6 (±60.3) MPa, to 927.1 (±60.4) and 1028.3 (±89.1) MPa at 24 h, 2 weeks and 4 weeks, respectively. A similar trend was observed for the compressive strength (going from 20.5 ± 7.1 to 31.8 ± 0.7 and 38.3 ± 0.7 over the different time points). The elastic modulus and strength of VSLA-NS were not statistically different to those of the sterilized cements at any time points (*p* > 0.51).

The flexural properties of vs. and VSLA-NS cements are shown in Figure 4. When submitted to the bending test, vs. cement bent until failure at 24 h and 6 weeks of setting. The bending modulus and bending strength of the unmodified cement were 3178.3 (±159.4) MPa and 58.5 (±4.8) MPa, respectively, at 24 h and decreased slightly (but not statistically) to 2903.7 (±53.2) MPa and 50.1 (±0.4) MPa at 6 weeks (*p* > 0.11). Unlike the control cement, the modified cement VSLA-NS showed high flexibility and bent without fracturing at 24 h, 2 and 4 weeks. From week 6, the specimens started to fracture upon bending. The bending modulus and bending strength of VSLA-NS cement increased over time until week 6 (from 1010 MPa and 14 MPa, respectively, at 24 h to 1466 MPa and 24 MPa, respectively, at 6 weeks).

The pullout data is presented in Figure 5. All cements were drillable and no differences in appearance between unmodified and modified cements were detected after screw pullout. The addition of LA (non-sterilized) to the formulation of vs. cement also reduced the pullout force (68% decrease). A similar trend was observed for the cements injected in sawbone, with a significant reduction in the holding strength of up to 72% (*p* < 0.001).

## 4. Discussion

Preparation of bone cement with bone-compliant mechanical properties for vertebral applications has become a recent focus in orthopaedic research. However, the development of such a cement, which also fulfills all other requirements of a functional cement in the application, is challenging. An ideal vertebral augmentation bone cement should be injectable with an adequate viscosity and easy to handle. Besides this, it should have a high radiopacity to facilitate detection of its placement in the vertebral body, adequate setting time (approx. 15 min) and low curing temperature. In addition to having bone-like mechanical properties, the cement should provide sufficient and immediate reinforcement of the vertebral body upon implantation, which does not deteriorate over time in vivo. It should also be able to provide sufficient reinforcement to hardware such as screws. Naturally, all material components also need to be sterilizable.

In the current study, we assessed the functional properties of a low-modulus PMMA bone cement containing 12 vol% sterilized or non-sterilized LA. The effect of sterilization on the composition of LA was analyzed using ^1^H NMR and SFC-MS/MS analysis. No structural differences was detected by ^1^H NMR, nor by the more sensitive SFC-MS/MS analysis [59,60,61]. Under thermal processing (>70 °C), FAs are usually unstable and prone to degradation via oxidation with a rate of degradation that correlates to the degree of unsaturation of the compounds [61,62]. However, Fidler et al. [59] investigated the effect of pasteurization and sterilization on the fatty acid composition in human milk using gas-liquid chromatography and found that exposing human milk to temperatures up to 120 °C for 20 min did not typically affect the composition of the fatty acids contained in the samples, although minor oxidative losses of polyunsaturated FA could occur. A degradation of LA might affect the functional properties of acrylic bone cements. However, the heat treatment used herein did not significantly influence LA’s ability to modify the properties of the cement, as verified by mechanical testing in compression as well as in the evaluation of handling properties and injectability. Indeed, the sterilized formulation had similar compressive strength and compressive modulus over time as the non-sterilized formulation. Besides modifying the mechanical properties of standard PMMA cement, LA allows for a significant reduction of the maximum polymerization temperature (by almost 40 °C) of the cements. This decrease in peak temperature due to the use of additives was also described by previous authors, using either LA [39,46] or other compounds [34,38,41,63]. Lower polymerization temperature is an important outcome, as it will minimize the risk of thermal necrosis of the neighboring tissues. In the present study, the setting time of unmodified cement was not significantly altered by the use of LA (sterilized and non-sterilized).

The additive LA also reduced the glass transition temperature of the modified formulation [46]. This finding was expected as the material becomes more flexible when LA, which acts as a plasticizer [39,64], is added. The mechanism of radical addition onto LA by the growing chain end in methyl methacrylate (MMA) polymerisation has been described previously [44,46,65]. The decrease in T*_g_* is likely also related to chain transfer by LA, resulting in shorter polymer chains as well as the presence of unreacted MMA. LA strongly affects the rate of polymerization by competing reactions to the regular addition of MMA to the growing chain end. LA contains hydrogen in allylic position, i.e., hydrogens in α-position to the double bonds, that are easily abstracted by initiator or growing chain ends radicals in the system rendering a resonance stabilized allylic radical. This radical is so stable that it cannot propagate, but instead terminates by combination with another radical to inhibit polymerization by so-called degradative chaintransfer [66].

Interestingly, post polymerization, the material slowly becomes harder upon storage. After two weeks, the T*_g_* increased (see Table 1), accompanied by an increase in modulus. During polymerization, the molecular motion is increasingly restricted as monomer is converted to polymer and also crosslinking occurs. Upon cooling, free volume is “frozen” into the material that now is in its glassy state. Slow molecular motions could, however, progressively reduce this free volume, densify the material and hence raise T*_g_* and modulus [67].

The use of LA had a positive effect on the injectability of the cement. It slowed down the polymerization reaction and as a result, increased the working time of the cements by prolonging the injection time [39]. 

The stiffness of both LA-modified cements was reduced (by up to 70%) after 24 h [44,46], matching that of healthy human vertebral cancellous bone (E = 10–976 MPa) [68,69]. The strength of the LA-modified cement after 24 h, 20.5 MPa, is well above that reported for trabecular bone [70], and approximately 10 times the pressures measured in intervertebral discs in vivo [71]. Despite the increase in both stiffness and strength over time, the stiffness of the LA-modified cements was still in the (upper) range of healthy vertebral cancellous bone [68,69]. 

The unmodified cement (VS) exhibited flexural properties in the range of regular acrylic bone cement [63,72], and in conformity with the requirements in ISO 5833 [50]. On the other hand, similarly to the compressive properties, the flexural properties were significantly affected by the introduction of LA. When tested in bending, these cements exhibited high flexibility and did not fail under bending until week 6. This can be explained by an initial hyperelastic behavior of the material, that over time changed due to the successive molecular motion leading to a denser, more plastic material, as mentioned previously. It can also be noted that the modified cement did not fail under compression either—the compressive strength reported as per the standard essentially corresponds to the yield stress, and the modified cements continued to deform under compressive stresses but did not break. The modified acrylic cement prepared herein exhibited lower flexural properties than most of the low-modulus cements described elsewhere [41,43,63], as well as those required for set cement according to the ISO standard—which stipulates 1800 MPa and 50 MPa, for the modulus and strength, respectively [50]. It should be noted however, that this standard was developed for cements used in joint replacement, and not for the spine. Jiang et al. [63] reported a decrease of the bending modulus and bending strength for three vertebroplastic PMMA bone cements modified with mineralized collagen. On average, these modified cements had a bending modulus and bending strength of 2100 MPa and 50 MPa, respectively, which is statistically lower than the respective standard cement but still in agreement with the requirements of the standard [50]. Harper et al. [43] observed an increase in bending modulus and bending strength for PEMA bone cements reinforced with hydroxyapatite particles from 835 to 1746 MPa and 29.3 to 43.3 MPa, respectively, as the amount of additives increased from 0 to 40 wt%, i.e., all in the flexural range required by the standard [50]. Gutiérrez-Mejía et al. [41], on the other hand, prepared a PMMA-based bone cement containing various amounts of core-shell nanoparticles. The cements therein had flexural properties lower than those established for set cement in the ISO standard, for example that with 20 wt% particles had a bending modulus and bending strength of 1610 MPa and 25 MPa, respectively, similar to the LA-modified cement at six weeks of storage. 

Despite the considerable decrease in bulk mechanical properties compared to the regular cement, the modified bone cements showed a pullout force comparable to the holding power of CaP cements [57,73]. Pujari-Palmer et al. [57] determined the pullout force of an experimental formulation of strong brushite cement injected into a similar system configuration (Sawbone^®^ with bovine cortical shell) to the one used in the present study. While the compressive strength of the CaP cements was considerably higher than the LA-modified acrylic bone cement presented herein, only slightly higher pullout strengths of 1000–1300 N were reached, likely due to the lower resistance to other loading modes of the CaP cements [74]. Stadelmann et al. [73] performed pullout tests from a less dense model of Sawbone^®^ (0.12 g/cm^−3^), and measured the pullout force of a commercial CaP cement (Hydroset^®^, Stryker Osteosynthesis, Selzach, Switzerland). Specimens with cortical fixation of 2 and 3 mm thickness and inserted at an augmentation depth of 15 mm, had a holding strength of 546 and 855 N, respectively, which is comparable to the VS-LA cement. The benefits provided by PMMA bone cement augmentation in improving the fixation strength of cancellous screws in severely osteoporotic bone has been widely described in the literature [75,76,77]. However, the drawbacks of PMMA bone cement, in particular the exothermic polymerization reaction and mismatch in stiffness between cement and cancellous bone, are still concerns, which may be resolved by using the present cement. 

There are some limitations to the present study. Firstly, the use of Sawbone^®^ rather than human osteoporotic bone limits the comparability with the clinical situation as Sawbone^®^ has a substantially different structure and mechanical properties than human bone. In addition, the presence of bone marrow might limit the penetration of the cement and thus affect the pullout strength of screws augmented with bone cement. Furthermore, the number of samples used to evaluate LA degradation and the handling properties was limited (n = 3 per group), although fulfilling the requirements of the standard for the latter properties [50].

In summary, the sterilization process did not significantly affect the composition of the additive LA, although slight degradation of the compound was observed. Besides having appropriate bone-compliant mechanical properties, the modified cement demonstrated adequate setting time and a reduced maximum polymerization temperature. It was injectable for more than 15 min and exhibited both high flexibility and a screw holding power similar to CaP cement. Similar formulations (LA-modified PMMA cements) have previously shown reinforcement of the vertebral body in an ex vivo study [26], and been found to give an adequate biological response in both in vitro and in vivo scenarios. [44,47]. The LA-modified cement evaluated herein could therefore be considered a good candidate for vertebral augmentation.

## 5. Conclusions

In this study we demonstrated that it is possible to prepare a fully sterile formulation of LA-modified PMMA bone cements by autoclaving the linoleic acid additive. The sterilized formulation displayed similar functional properties to the non-sterilized cement. In particular, it exhibited a compressive stiffness of 560 MPa, a compressive strength of 20.5 MPa, and a similar injectability to the non-sterilized LA-modified cement. The temperature generated during polymerization was significantly lower than that of unmodified cement (31.1 °C compared to 66.8 °C). In addition, LA-modified cement exhibited high flexibility when submitted to bending (flexural modulus of 1010 MPa) and a significant holding strength in a synthetic model of orthopaedic screw augmentation, comparable to CaP cements. The current findings suggest that (1) sterilization of the linoleic acid component by autoclaving does not significantly alter the properties of low-modulus cement; (2) LA-modified bone cements are a promising alternative for vertebral augmentation in osteoporotic patients or in other orthopaedic applications requiring the use of a low-modulus cement in combination with hardware. 

## Figures and Tables

**Table 1:** Curing properties of the control (VS) and LA-modified cements (Sterilized VSLA-S and Non-Sterilized VSLA-NS); n = 3 for each group.

Cement	t*_setting_* [Min]	t*_doughing_* [Min]	T*_max_* [°C]	T*_g_* [°C]	
				24 h	2 weeks
VS	22.1 (±1.0)	9.1 (±0.4)	66.8 (±2.7)	102.8 (±1.3)	114.0 (±2.5)
VSLA-NS	24.7 (±1.0)	12.3 (±0.3)	28.2 (±0.4)	74.7 (±4.8)	87.8 (±2.5)
VSLA-S	20.8 (±1.0)	9.9 (±0.1)	31.1 (±1.1)	78.0 (±3.3)	78.0 (±0.6)

**Table 2:** Compressive quasi-static mechanical properties of the control (VS) and LA-modified cements (Sterilized and Non-Sterilized LA); n = 6 for each group and time point. CS = Compressive Strength, in MPa, and E = elastic modulus, also in MPa.

	VS		VS-LA (NS)		VS-LA (S)	
Time Point	CS (±SD)	E (±SD)	CS (±SD)	E (±SD)	CS (±SD)	E (±SD)
24 h	100.7 (±3.1)	2140.4 (±128.8)	28.3 (±5.1)	494.7 (±51.8)	20.5 (±7.1)	559.6 (±60.3)
2 weeks	96.3 (±5.2)	2075.2 (±114.3)	30.5 (±0.8)	803.3 (±65.8)	31.8 (±0.7)	927.1 (±60.4)
4 weeks	91.5 (±16.5)	2070.0 (±103.1)	36.5 (±0.6)	947.8 (±64.4)	38.3 (±0.7)	1028.3 (±89.1)

## Data Availability

Not applicable.
